# Interactome based identification and validation of prefoldin 5-α for prognosing CNS leukemia in B-ALL patients

**DOI:** 10.1038/s41598-022-19489-7

**Published:** 2022-09-15

**Authors:** Tessy Xavier, Lakshmi Sumitra Vijayachandran, Rumamol Chandran, Ullas Mony, Anitha Augustine, Neeraj Sidharthan, Rema Ganapathy, Pavithran Keechilat, K R. Sundaram, Krishnakumar N. Menon

**Affiliations:** 1 Centre for Nanosciences and Molecular Medicine, Amrita Vishwa Vidyapeetham, Ponekkara, Kochi, 682 041 India; 2 Department of Medical Oncology and Department of Clinical Hematology, Amrita Institute of Medical Sciences and Research Centre, Amrita Vishwa Vidyapeetham, Kochi, Kerala India; 3 Department of Biostatistics, Amrita Institute of Medical Sciences and Research Centre, Amrita Vishwa Vidyapeetham, Kochi, Kerala India

**Keywords:** Prognostic markers, Protein-protein interaction networks, Biochemistry, Cancer, Biomarkers

## Abstract

We report here the identification and validation of prefoldin 5-alpha (PFDN5-α) for the first time as prognostic biomarker for prediction of central nervous system (CNS) leukemia of B cell acute lymphoblastic leukemia (B-ALL) origin. Since cerebrospinal fluid (CSF) cytology being the gold standard of diagnosis for CNS leukemia with poor sensitivity, mandatory prophylactic intrathecal chemotherapy is administered irrespective of patients develop CNS leukemia. Thus, using interactome studies, we identified PFDN5-α as a prognostic biomarker for predicting CNS leukemia by interacting lymphoblastic proteins and CSF from B-ALL patients using far-western clinical proteomics approach. Validation by both western and ELISA methods confirmed our results. For further clinical translation, we performed Receiver Operating Characteristic (ROC) curve analysis generated from CNS +ve (n = 25) and −ve (n = 40) CSF samples from B-ALL patients and identified PFDN5-α-CSF reactivity cut-off value as 0.456. Values below 0.456 indicate the patient is at risk of developing CNS leukemia and suggestive of having intrathecal chemotherapy. Further flow cytometry validation for CNS leukemia positivity revealed that with increasing blast cells, a decrease in PFDN5-α-CSF reactivity confirming ELISA based PFDN5α-CSF reactivity assay. Predicting CNS leukemia development risk by ELISA based PFDN5-α-CSF reactivity assay could have potential in the clinical management of CNS leukemia.

## Introduction

Central nervous system B-ALL^1^ poses major challenges in the clinic with 3–5% of B-ALL patients at diagnosis and 30–40% during relapse develop CNS leukemia^2^. Despite CSF cytology being the gold standard for diagnosing CNS leukemia^3,4^, the poor sensitivity results in mandatory prophylactic intrathecal chemotherapy irrespective of patients developing CNS leukemia with substantial neurological consequences^5,6^. Thus, discovering prognostic markers towards predicting CNS leukemia is essential. Despite polymerase chain reaction and flow cytometric evaluation of CSF could identify 50% of CNS leukemia in B-ALL patients at presentation, lack of clinically correlative standardized data makes CNS leukemia prediction challenging^2^. Similarly, T-cell immunophenotype and cytogenetic abnormalities also failed to predict CNS involvement^7,8^. However, different molecular interactions seen in CNS leukemia^9–11^ could be explored towards identifying prognostic markers. Therefore, we hypothesized that sub-microscopic disease specific biochemical CSF entities may interact with lymphoblastic proteins and these interactions could assist in discovering potential markers for predicting CNS leukemia. Though the daunting challenge of identifying interactions between two unknown molecules belonging to CSF and lymphoblastic proteins exists, the established far-western interactome technology was effective in deciphering molecules belonging to such interactions^12^. Thus, lymphoblastic proteins and CSF components of B-ALL patients at CNS positivity (presentation/relapse) and at remission (CNS negative) were interacted using far-western technique to obtain an interactome profile to identify specific interactions of clinical significance. In this report, we identified the protein PFDN5-α and validated its prognostic significance in predicting CNS leukemia in B-ALL patients. Further, we derived a cut-off value to predict the risk of developing CNS leukemia that prophylactic intrathecal chemotherapy could be avoided.

## Materials and methods

### Materials

Urea and Thiourea, Bromophenol blue, Tris-base, Ammonium persulfate, Bradford protein assay reagent CHAPS, Iodoacetamide, DTT, E64, IPTG, Sodium carbonate, Sodium bicarbonate, TMB, DMSO, Aprotinin, Tryptone, SDS, Ni-agarose beads, Trypsin and Trypsin reaction buffer were from Sigma-Aldrich, USA. ASB-14-4 (tetradecanoylamidopropyl dimethyl ammonio-butane sulfonate, (Calbiochem, USA), Ampholyte (pH 3–10), IPG strips pH 3–10 and Mineral oil (biotechnology grade) (Bio-Rad), TEMED, Ethanol (Fisher Scientific, USA), Methanol (Merck, USA). Penicillin/streptomycin, Hybridoma serum free medium and Fetal bovine serum (FBS) and TOP10 from Invitrogen, USA. Protogel (National Diagnostics, USA), Amicon ultra-4 centrifugal filter unit, PVDF (Millipore, USA), Streptavidin-HRP conjugate Biotin (Thermo Scientific, USA), X-ray film and X-ray Cassette (Kodak, India), Developer and Fixer (Premier, India), Acetonitrile and Ammonium bicarbonate (Fluka, USA), ESI-Q-TOF (Bruker, Germany), Ampicillin and Kanamycin (Hi-media, India), pCMV-XL5- gene construct of PFDN5α, CIP29, ECH1 and PRDX6 (Origene, USA). EcoRI, NotI, DNA ladder and T4 Ligase (TaKaRa, India), QIAquick kit (Qiagen, India), BL21 (Merk-Millipore, USA), pET28a vector (Merk-Millipore, USA), DH5α (ATCC, USA), Ammonium acetate, NaCl, Tween 20, Acetone, Yeast extract, Disodium hydrogen phosphate and HCl (Nice chemicals, India), Multiplate reader (BioTek, USA).

### B-ALL patient CSF samples

The study was conducted according to the guidelines of the Declaration of Helsinki, and approved by the Institutional Ethics Committee of Amrita Institute of Medical Sciences (IEC-AIMS-2016 Dated 25-01-2016). CSF samples from B-ALL patients were collected at CNS +ve (presentation/relapse positive for blast cells)/CNS –ve remission status with written informed consent from all subjects involved in the study according to institutional human ethics committee guidelines with approval. CSF includes sequential samples from B-ALL patients of various age groups, both genders with different cytogenetics abnormalities and treatments (Table S1).

### CSF sample processing and biotinylation

The CSF samples obtained from the same (sequential samples) and different patients were spun down (800*g* for 5 min at 4 °C) to remove any cells and were kept as aliquots at − 80 °C until processed. CSF biotinylation were carried out with 20 mM biotin (10% v/v) to CSF for 3.5 h at room temperature and then stored at − 20 °C as per Menon et al.^12^.

### Cell culture and cell lysate preparation

The B-lymphoblastic JM-1 cell line was obtained from National Centre for Cell Sciences, Pune, India (originally from ATCC, USA). Cells were cultured in serum-free hybridoma medium containing 10% FBS and 1% penicillin/streptomycin at 37 °C in 5% CO_2_ incubator as described in Xavier et al.^13^. Cells were trypsin treated and spun down at 800*g* for 8 min. The pellet was PBS washed and lysed in 8 M urea–2 M thiourea solution with protease inhibitors E64 and Aprotinin. Further, presence of any particulate material in the lysate was dispersed by sonication and concentrated using Amicon ultra-4 centrifugal filter unit at 4500*g* for 45 min at 4 °C and washed five times with 8 M urea and then used for 2D PAGE profiling^13^.

### Protein estimation

Protein estimation of concentrated cell lysate was assayed using Bradford reagent and absorbance was measured at 595 nm using micro plate reader.

### Two-dimensional polyacrylamide gel electrophoresis (2D-PAGE)

2D-PAGE was performed essentially the same way as described in Xavier et al.^13^. Briefly, 200 µg total proteins from lysate sample were taken in 125 µL of rehydration buffer and rehydrated actively at 50 V/20 °C for 18 h using IEF cell (Bio-Rad). After rehydration, the strip was subjected to IEF as described before^12,13^. This is followed by incubation in equilibration buffer (50 mM Tris, pH 8.8, 6 M urea, 30% glycerol, 2% SDS) with 0.3% DTT for 10 min. Subsequently, incubated with 4.5% iodoacetamide containing equilibration buffer for 10 min and subjected to SDS-PAGE. Proteins resolved in the gel were visualized by silver staining or used for western blotting^12^.

### 2D-Far-western analysis using CSF

2D-PAGE was performed using 200 µg total proteins as described above^13^. For far western, biotinylated CSF sample (2.0 µg concentration CSF proteins in 1 mL of 5% gelatin at 4 °C overnight) from patient was probed against lymphoblastic proteins on a 2D western blot. The blots were washed 3 times 10 min each in TBST (Tris Buffered saline + 0.2% Tween 20) and subjected to streptavidin-HRP staining (1 in 3000 dilutions with 5% milk in TBST). The chemiluminescence signal following CSF reactivity to protein spots was captured on X-ray film. Subsequently, the CSF reactivity pattern obtained with CNS +ve at presentation was compared with sequential CSF samples reactivity pattern from the same patient collected at different periods of remission respectively. The procedure was repeated for other patient CSF samples. The sequential samples from patients varied in time frame from 3 weeks to 1.5 years. The intra and inter patient reactivity pattern analysis between three patients resulted in identification of 4 specific spots with consistent reactivity to CNS +ve/−ve CSF.

### Quantification of CSF reactive spots on 2D blots by Image J software

**Figure 1:**
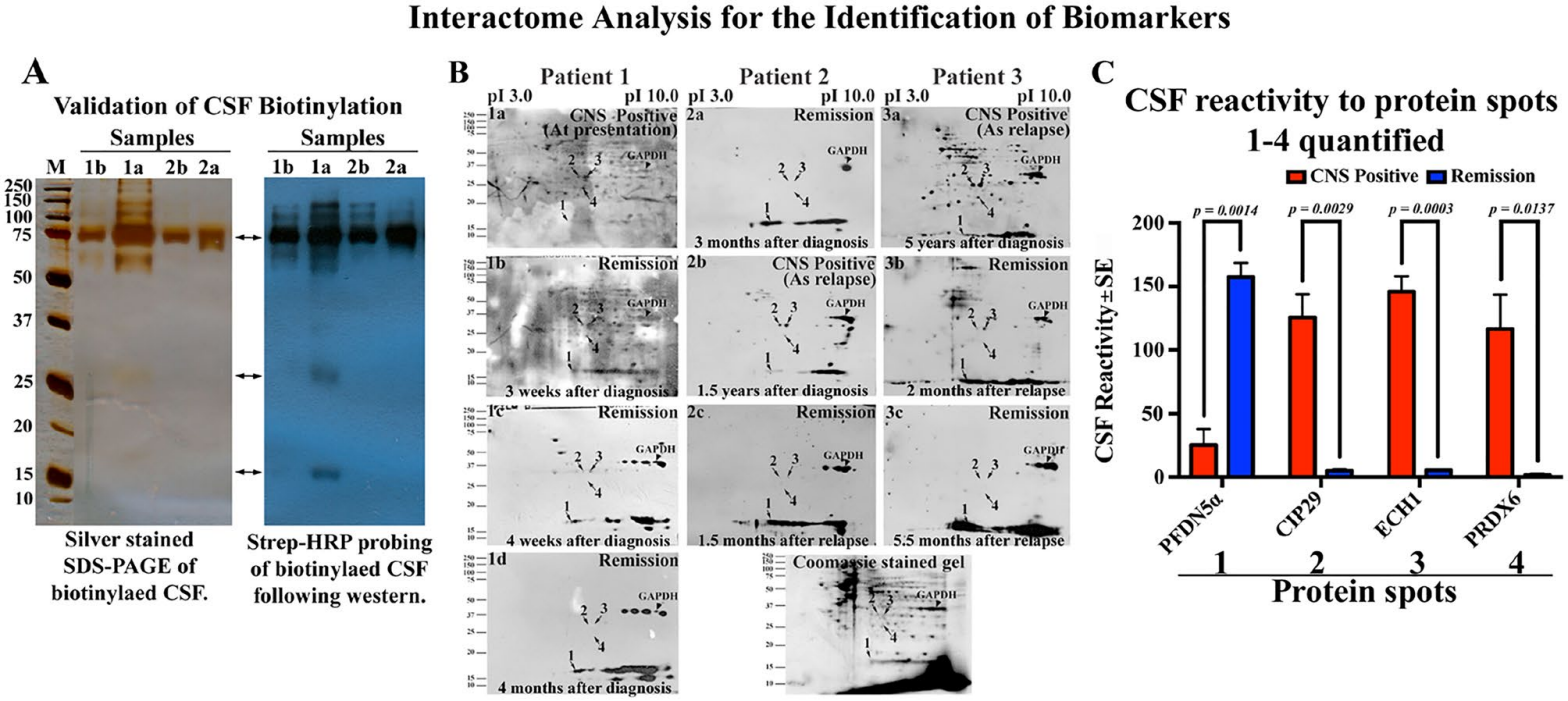
Identification of specific CNS +ve and −ve B-ALL CSF reactivity to lymphoblastic proteins using 2D far-western interactome analysis. (**A**) Validation of the extent of biotinylation of CSF proteins using 1 µg (left panel) or 0.25 µg (right panel) of CSF samples from patients 1 and 2 resolved in 12% SDS-PAGE. Despite the difference in the quantity of protein load, the streptavidin-HRP probed patterns on the blot (right panel) were similar (double headed arrows) to that of silver stained gel (left panel) indicating that the silver stained biotinylated proteins in the gel when probed with streptavidin-HRP were getting visualized on the blot highlighting the sensitivity following biotinylation and the advantage of small quantity of sample for performing far-western. (**B**) Pre B-ALL cell line (JM-1) protein lysate was prepared to obtain the proteomic profile of B-ALL proteins by two dimensional SDS-PAGE profiling and western blotting onto PVDF membrane. Far-western analysis was performed by probing profiled on PVDF membrane with biotinylated CSF samples from CNS +ve and −ve (remission) patients. The CSF reactivity pattern of sequential CSF samples (a–d) from the same patient from CNS +ve (at presentation or relapse) and CNS −ve (remission) from different time points were compared among and between patients 1–3. The protein spots that are common and unique which showed differential CSF reactivity in a consistent way in CNS +ve and −ve cases are marked with arrows (spots 1–4). Consistently CSF reacting protein spots 1–4 were selected for mass spectrometric analysis from the corresponding coommassie stained gel (**A**) to decipher the protein identity. The internal control GAPDH is shown by arrowheads. (**C**) Quantified CSF reactivity on the 2D blot (values of spots in 1a, 2b and 3a for CNS +ve and 1d, 2c and 3c for CNS −ve; n = 3) were found to be significantly different between CNS +ve and −ve cases. The protein identity of spots 1–4 was found to be PFDN5α, CIP29, ECH1 and PRDX6 respectively (Table S2).

The CSF reactive spots corresponding to the spots marked in Fig. 1B was measured using the Image J software by making a selection on the spot. Then the value of the background of the same blot was subtracted from the value of the spot. The internal control spot corresponding to GAPDH which showed reactivity irrespective of CNS positivity and remission was also measured in the same way as mentioned above and the value corresponding to GAPDH was used for normalizing the value of these differentially reactive spots. The normalization was done by dividing value of the spot/value of GAPDH multiplied by average value of GAPDH from different blots.

### Mass spectrometric analysis of CSF reactive lymphoblastic proteins

Based on the comparison of the CSF reactivity profile towards proteins of lymphoblastic origin on a 2D blot, the protein spots which showed differential reactivity to CSF (spot 1–4) were cut out from the Coomassie stained gel and de-stained with 100 mM ammonium bicarbonate/Acetonitrile (1:1 vol/vol) solution and spun down^12^. The gel piece was dehydrated by incubation for 30 min at room temperature in 100% acetonitrile with occasional vortexing. Then the gel piece was dried in vacuum and ice cold trypsin reaction buffer was added to cover the gel piece. The mixture was incubated at 37 °C for overnight till the gel piece becomes saturated with trypsin. The supernatant containing digested protein in the spot was collected. The proteins were identified by mass spectroscopy analysis using ESI Q-TOF mass spectrometry^12^.

### Cloning and expression of identified proteins using bacterial expression system

The pCMV6-XL5-PFDN5α was transformed to DH5α competent cells for plasmid amplification and subsequently purified by alkaline lysis method. The gene insert (PFDN5α) was excised from pCMV6-XL5 vector and ligated to pET28a bacterial expression vector. Subsequently transformed into BL21 strain and optimized the conditions for expression of protein/s.

### Expression and purification of recombinant PFDN5α from bacteria

200 mL culture of transformed BL21 (with pET28a-PFDN5α) was induced using 0.5 mM IPTG at 37 °C for protein production. After 5 h of induction, cells were pelleted down, lysed in lysis buffer of pH 8.0 (8 M Urea, 50 mM Tris-Base, 500 mM disodium hydrogen phosphate) containing protease inhibitors, sonicated to remove the aggregates and centrifuged at 14,000 rpm for 5 min. Supernatant containing the expressed protein was collected and subjected to Ni-NTA column purification as described by the manufacturer Sigma.

### Validation and translation of B-ALL CSF reactivity to PFDN5α for prognostication of CNS leukemia

#### ELISA based assay

The purified recombinant PFDN5α (2 µg) was western blotted to PVDF membrane and probed with biotinylated CSF samples from B-ALL patients as before and the reactivity was quantified using Image J software. For ELISA, 300 ng of purified recombinant PFDN5α protein coated on 96 well plates were incubated with 35 ng of biotinylated CSF samples from the patients for 1 h at room temperature. Washed three times with TBST (TBS with 0.05% Tween 20) and incubated with Streptavidin HRP for 1 h at room temperature (1/7000 dilutions in 1% BSA). Washed in TBST for three times and incubated with HRP substrate TMB for 10 min. The reaction was stopped by adding 1 N HCl and the color was measured at 450 nm.

#### Flow cytometry-based assay

A minimum of 1 mL of CSF was collected in a tube and processed within 24 h of collection. CSF was centrifuged at 800*g* for 5 min at 4 °C. The supernatant was stored at − 80 °C for PFDN5α ELISA validation and the cells were resuspended in 100 µL of remaining CSF. The antibody combination was added to the tubes for immunostaining. After staining 2 mL sheath fluid (BD) was added to the tube and centrifuged at 2500 rpm for 3 min, resuspended the pellet in 100 µL of sheath fluid, and acquired immediately on BD FACS Canto II. The tubes were acquired until empty to collect all the events. After acquisition the debris were excluded from the events by setting up the forward scatter and side scatter threshold. For B-ALL, cells were identified using CD45 plots. A gate was generated using CD19 and CD34 antibody channels and events showing CD19/CD34 positive cell cluster as well as CD34 alone positive cells were considered as blast/aberrant phenotypes.

### Statistical analysis

Unpaired two-tailed Students *t* test assuming equal variances utilizing Graphpad Prism were carried out and statistical significance were set at p ≤ 0.05 and represented with star (*). Receiver operating characteristic curve (ROC) analysis was performed using SPSS software version 22.0 to identify a cut off value to predict CNS positivity in B-ALL patients. For flow cytometry-based samples and respective ELISA, one tailed unpaired analysis was carried out with unequal variance. For flow cytometry-based samples and respective ELISA, one tailed unpaired analysis was carried out with unequal variance.

## Results

### Identification of specific B-ALL-CSF reacting protein spots by 2D-far-western interactome analysis

At first, CSF proteins were biotinylated^12^. Since biotin forms covalent bonds with primary amines of CSF proteins, unified protein tagging was achieved demonstrated by detection of the same proteins bands resolved in SDS-PAGE by silver staining and streptavidin-HRP staining on far-western blots following SDS-PAGE (Fig. 1A).

Further, far-western analysis was performed by interacting biotinylated CSF from CNS +ve/−ve samples with 2D profiled JM-1 pre-B lymphoblast cell line proteins^12,13^ to detect CSF interacting lymphoblastic proteins (Fig. 1B). Comparison of CNS +ve/−ve CSF reactivity to lymphoblastic proteins from 3 different patients with their sequential samples at each visit (comprising 10 different CSF samples) led to identification of four specific proteins spots that showed consistent CSF reactivity to CNS +ve/−ve samples (Fig. 1B, arrows) despite heterogeneity in terms of age, sex, blast percentage, difference in duration between CNS positivity and remission and cytogenetic abnormalities (Table S1). Quantification of CSF reactivity spots following normalization against GAPDH revealed statistically significant low reactivity to spot 1 for CNS +ve CSF compared to CNS −ve (Fig. 1B,C; *p* = 0.0014). In contrast, proteins spots 2, 3 and 4 showed significantly high reactivity with CNS +ve CSF compared to CNS −ve (Fig. 1C; p values 0.0029, 0.0003, 0.0137).

### Identification and validation of B-ALL-CSF reactive spots-1–4 obtained following interactome analysis

**Figure 2:**
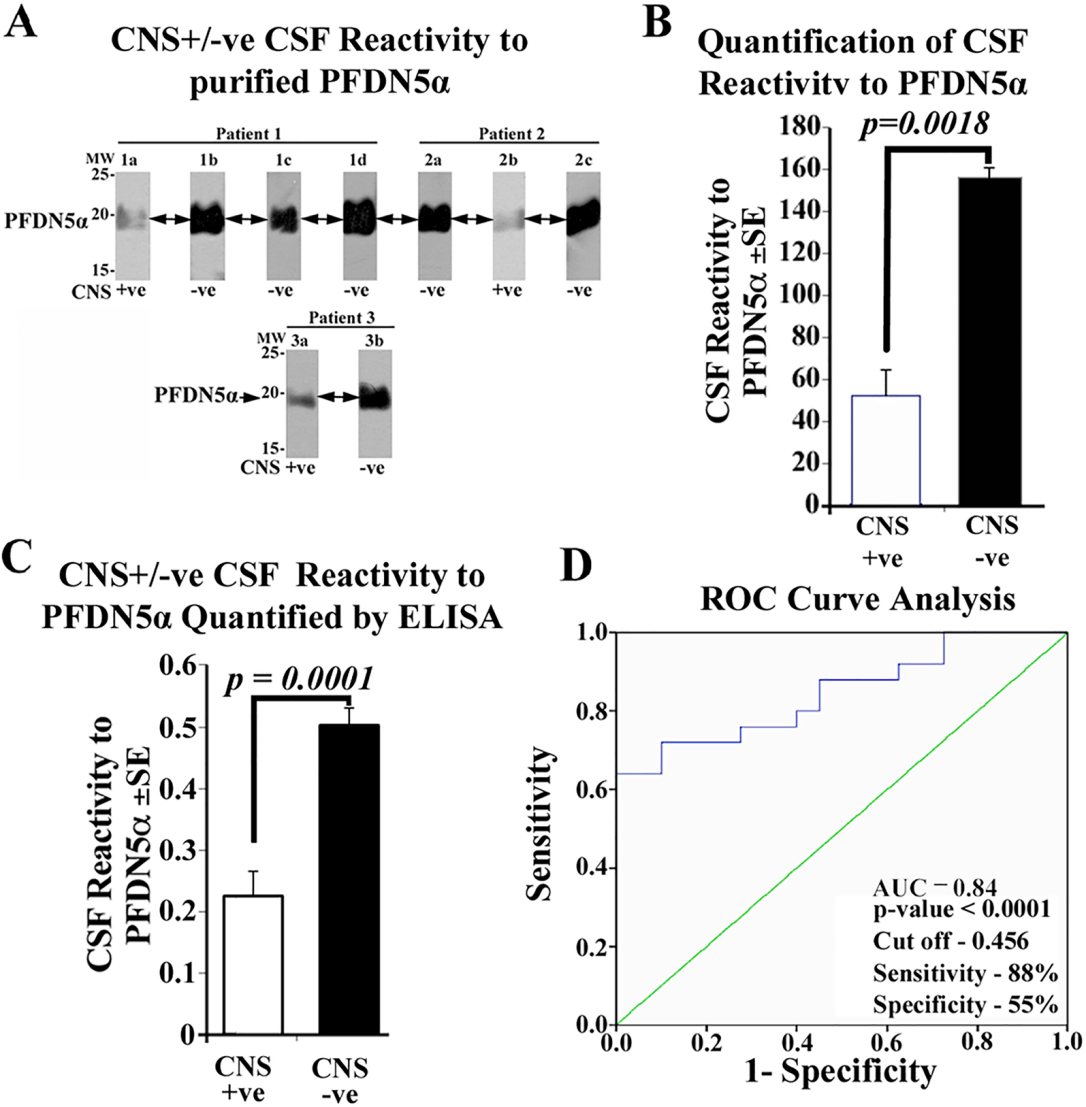
Validation analysis and quantification of CSF reactivity to purified recombinant PFDN5α using 1D western and ELISA platforms for determination of cut-off value by ROC curve analysis for prognostication. (**A**) Western blots showing validation of differential CSF reactivity of CNS +ve (presentation/relapse) and CNS −ve (remission) sequential samples from patients 1, 2 and 3 towards purified recombinant PFDN5α. The pattern of CSF reactivity validates to that observed on the 2D far western blot (Fig. 1A). (**B**) Quantification of CNS +ve [51.25 ± 23.23; n = 3; from 1a, 2b and 3a) and −ve (156.3 ± 8.27; n = 3; from 1d, 2c and 3b) CSF reactivity to purified PFDN5α protein showing statistical significance (*p = 0.0018; CNS +ve vs −ve). (**C**) Further validation and quantification of CSF reactivity of CNS +ve (n = 25) and −ve (n = 40) CSF samples to purified PFDN5α by ELISA showed statistically significant difference between CNS +ve (0.225 ± 0.202) and −ve (0.502 ± 0.177) CSF samples (*p* = 0.0001). (**D**) Receiver operating characteristic (ROC) curve analysis of reactivity to PFDN5α towards CNS +ve and −ve CSF samples to identify cut-off value to predict the CNS positivity in patients. The ROC curve plot shows that the area under the curve is 0.84 (95% CI 0.74–0.95; *p* = 0.0001) and the cut-off value at 88% sensitivity and 55.0% specificity is 0.456.

Based on the above interactome analysis showing promising difference in reactivity between CNS +ve and CNS −ve B-ALL CSF samples, protein spots 1–4 was excised out and subjected to mass spectrometry analysis^12^. The spots 1–4 was identified as PFDN5α, CIP29, ECH1 and PRDX6 respectively (Table S2). Among these proteins, PFDN5α was chosen for further validation analysis due to its established repressive role in controlling the oncogene cMyc involved in both hematopoiesis and leukemia. Thus, we purified recombinant his tagged PFDN5α following expression in bacterial expression system (Fig. S1). Using purified PFDN5α, we performed 1D western analysis with CNS +/−ve biotinylated CSF samples. Interestingly, as shown in Fig. 1B (1a vs 1d; 2a vs 2b and 3a vs 3c), PFDN5α (spot-1) showed consistently low CSF reactivity to CNS +ve and high reactivity to CNS −ve (remission) CSF samples (Fig. 2A).

This observation is similar to the results of 2D-far-western blots seen in Fig. 1B (spot 1 arrows in blots 1a vs 1d; 2a vs 2b and 3a vs 3c). Notably, a statistically significant difference between CNS +ve vs −ve B-ALL CSF sample is observed (*p* = 0.0018, Fig. 2B). The differential CSF reactivity to PFDN5α between CNS +ve/−ve CSF by 1D western validates and confirms the prognostic significance of PFDN5α in CNS leukemia. Thus, PFDN5α was taken for further validation for its potential use as a prognostic marker for predicting CNS leukemia.

### Consolidation of prognostic significance of PFDN5α by ELISA

To further consolidate the significance of CSF-PFDN5α reactivity and to identify a cut-off value useful to predict CNS leukemia, we tested the CNS +ve/−ve B-ALL CSF reactivity using alternative ELISA platform. A total of 25 CNS +ve and 40 CNS −ve B-ALL CSF patient samples were evaluated for its differential CSF-PFDN5α interaction between CNS +ve and CNS −ve CSF patient samples and plotted to identify a cut-off value for CNS leukemia prediction (Fig. 2C,D and Table S4, S4A, S4B). The CNS +ve and CNS −ve B-ALL CSF samples showed a quantitative average of 0.225 ± 0.040 and 0.502 ± 0.027 respectively with statistical significance *p* = 0.0001 (Fig. 2C). ROC curve analysis revealed an area under the curve (AUC) value of 0.84 showing the utility of ROC curve in discriminating between CNS +ve/−ve patients (Fig. 2D). The cut-off value was derived as 0.456 at 88% sensitivity (22/25) and 55.0% specificity (22/40) (Table S5) towards predicting CNS leukemia.

### Correlation of flow cytometry-based CNS positivity with CSF reactivity to PFDN5α by ELISA

**Figure 3:**
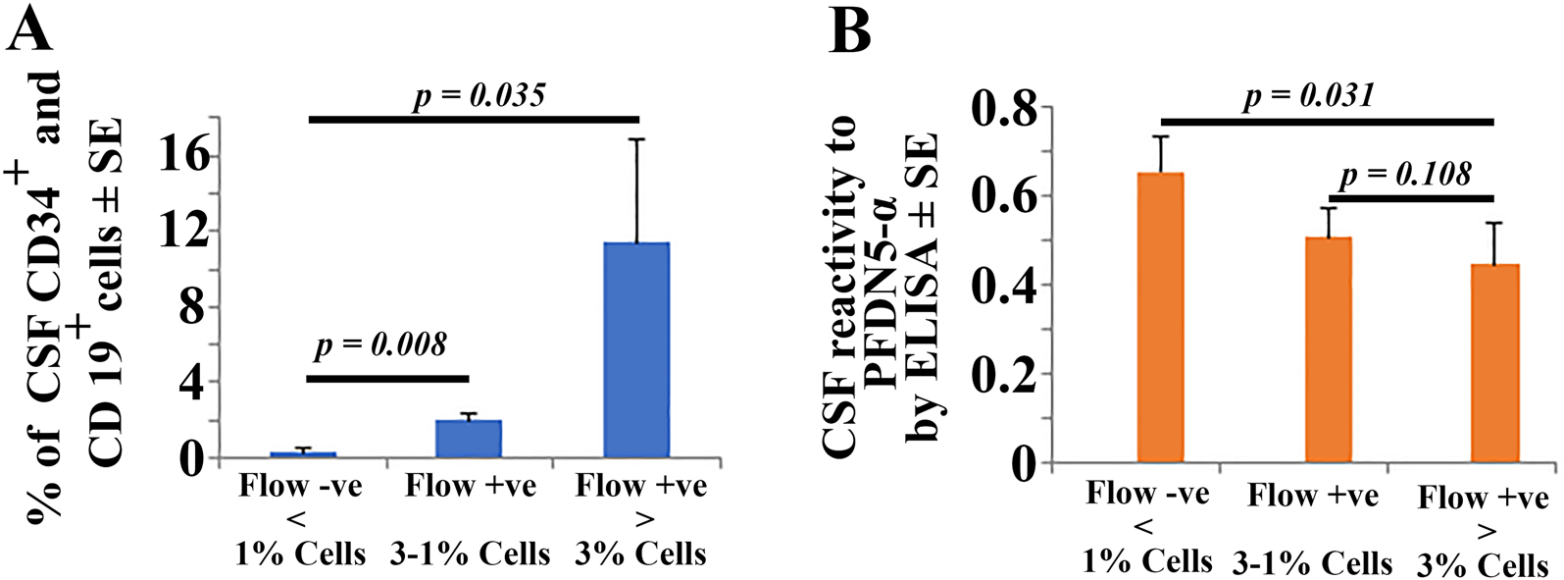
Validation Flow cytometry-based quantification of CD34^+^/CD19^+^ cells in the CSF following initial gating with CD45^+^ and the extent of CSF reactivity of the same samples to PFDN5-α by ELISA quantification. Note that with increasing number of CD34^+^/CD19^+^ cells (**A**), there is gradual decrease in reactivity of CSF to PFDN5-α identified by ELISA (**B**). More importantly, the CSF samples in which > 3% blast cells seen are statistically significant compared to CNS leukemia negative samples identified by flow (less than 1% CD34^+^/CD19^+^ cells) and that the average CSF reactivity to PFDN5-α is 0.448 ± 0.092, a value below the cut off value of 0.456 identified by ROC curve analysis.

Based on the ELISA reactivity to PFDN5-α, we verified whether flow cytometric approach of determining CNS positivity in terms of cell number correlates with ELISA based CSF reactivity to PFDN5-α. All the cases used in this study were negative by cytomorphology. We categorized the samples as flow positive or negative based on two criteria. The samples are considered as flow positive^14,15^ if there is discrete clonal population of cells with both CD19^+^/CD34^+^ with percentage of blast population should be ≥ 1% and below this value is considered negative by flow. In order to perform flow-based analysis, a total of seventeen B-ALL patient samples that are negative for CNS leukemia as per CSF cytomorphology were collected. Fourteen samples met our criteria and considered as flow positive and the remaining 3 were considered flow negative. All CSF samples were validated for their reactivity to PFDN5-α using ELISA quantitative assay as described in our manuscript. Following analysis by flow cytometry, we found that with increasing number of cells that are CD34^+^ and CD19^+^, the CSF reactivity to PFDN5-α decreases (Table S6 and Fig. 3). Note that the average cut off value by ELISA PFDN5-α CSF reactivity from 11 out of the 14 positive flow samples was found to be 0.448 ± 0.092. This value remained below the cut off value of 0.456. The three samples with % blasts between 1–3 showed higher CSF reactivity of 0.509 ± 0.066 to PFDN5-α indicating that ELISA interactome assay followed a similar pattern according to the cell number found in the CSF as per flow cytomorphology. Importantly, the three CNS negative samples by flow for CNS leukemia has an ELISA PFDN5α reactivity of 0.656 ± 0.075. This value is way above the cut off value of 0.456. These observations clearly show that ELISA based PFDN5α CSF reactivity clearly follow a pattern similar to that of flow results.

## Discussion

In the quest towards identifying a potential biomarker for predicting CNS leukemia in B-ALL patients, we used our established far-western clinical proteomics approach^12^ and identified four spots that showed differential CSF reactivity to CNS +ve/−ve B-ALL CSF among the many protein spots profiled on 2D-western blot [Fig. 1B (1a vs 1d; 2a vs 2b and 3a vs 3c) and C] The differential CSF reactivity against these four spots was significant between CNS +ve/−ve B-ALL CSF samples (Fig. 1B,C). These four proteins were identified as PFDN5α, CIP29, ECH1 and PRDX6 respectively. Among these four proteins, PFDN5α was further taken for validation to confirm its reactivity to B-ALL CSF using both 1D western and ELISA platform based on its potential role in controlling one of the genes involved in leukemia pathogenesis, the c Myc at different levels such as (a) repressing transcriptional activity of c-Myc, (b) promotes degradation of c-Myc, (c) transcriptional repression of Wnt4 involved in the Wnt-β-catenin pathway that activate c-Myc expression (Table S3)^16,17^. Moreover, point mutations in PFDN5α lead to abolition of above activities and are associated with leukemia and lymphoma^16^. Thus, validation of PFDN5α-CSF reactivity is crucial in proving its role as a prognostic marker. Our results on validation analysis clearly show that PFDN5α could serve as a potential prognostic marker in predicting CNS leukemia in B-ALL patients following 1D western analysis against purified recombinant proteins (Fig. 2A, Fig. S2). The data is consistent with 2D far-western results (Fig. 1B,C). In fact, prefoldins (PFDN1-6) have been found to serve as poor prognostic markers in gastric cancer^16,18^. Thus, PFDN5α-CSF interactions in B-ALL could turn out to be an important tool in the prognostication of CNS leukemia and reduce the burden and side effects associated with prophylactic intrathecal chemotherapy to some extent. In order to further develop an assay, and identify a cut-off value for prediction of risk of a B-ALL patient developing CNS leukemia in addition to its validation, we further assessed PFDN5α-CSF reactivity using ELISA platform (Fig. 2C,D and Table S4, S4A, S4B). The results were once again consistent with our 2D far-western (Fig. 1B,C and 1D western results (Fig. 2A) thereby consolidating the 2D-far western and 1D western finding. ROC curve analysis following ELISA assay identified a cut-off value of 0.456 to predict the risk of B-ALL patients developing CNS leukemia. Values below 0.456 cut-off indicate an underlying CNS involvement suggestive of intrathecal chemotherapy and values above indicates better prognosis and could be spared from intrathecal chemotherapy. Flow cytometric analysis shows that despite using entirely different approach measuring atypical cells by flow cytometry or a molecule by ELISA based interactome analysis, same conclusion is drawn (Fig. S2). This observation is in agreement with our previous analysis that CNS positive samples with significant proportions of cells (by CSF cytomorphology) showed low CSF reactivity to PFDN5-α. Based on the ROC curve, a value above the cut-off value of 0.456 is considered not requiring chemotherapy. Note that the average cut off value by ELISA based PFDN5-α CSF reactivity from 11 out of the 14 positive flow samples was found to be 0.448 ± 0.092. This value remained below the cut off value of 0.456 indicating high risk category. The other three positive flow samples showed % blasts below 3 and demonstrated higher CSF reactivity of 0.509 ± 0.066 to PFDN5-α indicating that ELISA interactome assay followed a similar pattern according to the cell number found in the CSF as per flow cytomorphology. Although the three samples in terms of sample number is low, the flowcytometry value indicates that patients were CNS negative for CNS leukemia and showed an ELISA PFDN5α CSF reactivity of 0.656 ± 0.075 suggestive of a correlation to the flow cytometry finding. These observations show that ELISA based PFDN5α CSF reactivity clearly follow a pattern similar to that of flow results. It is noteworthy that two entirely different approaches for CNS positivity measurements giving the same results indicating the increase confidence of this assay in prognosticating CNS leukemia of B-ALL origin.

Another important observation we noticed was the PFDN5-α ELISA assay is not dependent on the leukemia cell concentration in the CSF (Table S6). CNS leukemia prediction using PFDN5-α could bring in significant improvement in the clinical management of the disease and could avoid unnecessary prophylactic chemo burden to patients.

## Supplementary Information

Supplementary Information.

## References

[1] Terwilliger T, Abdul-Hay M (2017). Acute lymphoblastic leukemia: A comprehensive review and 2017 update. Blood Cancer J..

[2] Lenk L, Alsadeq A, Schewe DM (2020). Involvement of the central nervous system in acute lymphoblastic leukemia: Opinions on molecular mechanisms and clinical implications based on recent data. Cancer Metastasis Rev..

[3] Bigner SH (1992). Cerebrospinal fluid (CSF) cytology: Current status and diagnostic applications. J. Neuropathol. Exp. Neurol..

[4] Bürger B, Zimmermann M, Mann G, Kühl J, Löning L, Riehm H, Reiter A, Schrappe M (2003). Diagnostic cerebrospinal fluid examination in children with acute lymphoblastic leukemia: Significance of low leukocyte counts with blasts or traumatic lumbar puncture. J. Clin. Oncol..

[5] Surapaneni UR, Cortes JE, Thomas D, O’Brien S, Giles FJ, Koller C, Faderl S, Kantarjian H (2002). Central nervous system relapse in adults with acute lymphoblastic leukemia. Cancer.

[6] Jabbour E, Thomas D, Cortes J, Kantarjian HM, O’Brien S (2010). Central nervous system prophylaxis in adults with acute lymphoblastic leukemia: Current and emerging therapies. Cancer.

[7] Faderl S, Kantarjian HM, Talpaz M, Estrov Z (1998). Clinical significance of cytogenetic abnormalities in adult acute lymphoblastic leukemia. Blood.

[8] Frishman-Levy L, Izraeli S (2017). Advances in understanding the pathogenesis of CNS acute lymphoblastic leukaemia and potential for therapy. Br. J. Haematol..

[9] Buonamici S, Trimarchi T, Ruocco MG, Reavie L, Cathelin S, Mar BG, Klinakis A, Lukyanov Y, Tseng JC, Sen F (2009). CCR7 signalling as an essential regulator of CNS infiltration in T-cell leukaemia. Nature.

[10] Holland M, Castro FV, Alexander S, Smith D, Liu J, Walker M, Bitton D, Mulryan K, Ashton G, Blaylock M (2011). RAC2, AEP, and ICAM1 expression are associated with CNS disease in a mouse model of pre-B childhood acute lymphoblastic leukemia. Blood.

[11] Vidal M, Cusick ME, Barabási AL (2011). Interactome networks and human disease. Cell.

[12] Menon, K. N., Steer, D. L., Short, M., Petratos, S., Smith, I., Bernard, C. C. A. A novel unbiased proteomic approach to detect the reactivity of cerebrospinal fluid in neurological diseases. *Mol. Cell. Proteom.* **10** (2011). 10.1074/mcp.M110.000042 PMC3108827 21421798

[13] Xavier T, Ganesan TS, Menon KN (2010). A simple and efficient method for processing of cell lysates for two-dimensional gel electrophoresis. Electrophoresis.

[14] Thastrup M, Marquart HV, Levinsen M (2018). Central nervous system involvement detected by flow cytometry is a risk factor for relapse in childhood acute lymphoblastic leukemia. Blood.

[15] Craig FE, Ohori NP, Gorrill TS (2011). Flow cytometric immunophenotyping of cerebrospinal fluid specimens. Am. J. Clin. Pathol..

[16] Fujioka Y, Taira T, Maeda Y, Tanaka S, Nishihara H, Iguchi-Ariga SMM, Nagashima K, Ariga H (2001). NM-1, a c-Myc-binding protein, is a candidate for a tumor suppressor in leukemia/lymphoma and tongue cancer. J. Biol. Chem..

[17] Millán-Zambrano G, Chávez S (2014). Nuclear functions of prefoldin. Open Biol..

[18] Yesseyeva G, Aikemu B, Hong H, Yu C, Dong F, Sun J, Zang L, Zheng M, Ma J (2020). Prefoldin subunits (PFDN1-6) serve as poor prognostic markers in gastric cancer. Biosci. Rep..

