# Supplementary material for: Interactome based identification and validation of prefoldin 5-α for prognosing CNS leukemia in B-ALL patients
Source: Sci Rep. 2022 Sep 15;12:15491. doi: 10.1038/s41598-022-19489-7 (PMC9477816; doi:10.1038/s41598-022-19489-7)
Supplement: Supplementary file 1 — Supplementary Information. [file 41598_2022_19489_MOESM1_ESM.pdf]

INTERACTOME BASED IDENTIFICATION AND VALIDATION OF PREFOLDIN 5-α FOR PROGNOSING CNS LEUKEMIA IN B-ALL PATIENTS

Tessy Xavier[†], Lakshmi Sumitra Vijayachandran[†], Rumamol Chandran[†], Ullas Mony[†], Anitha Augustine[†], Neeraj Sidharthan[^], Rema Ganapathy[^], Pavithran Keechilat[^], Sundaram K R[#] and Krishnakumar N Menon[†]*

Centre for Nanosciences and Molecular Medicine[†]

Amrita Vishwa Vidyapeetham

Ponekkara, Kochi-682 041

Department of Medical Oncology and Department of Clinical Hematology[^]

Department of Biostatistics[#]

Amrita Institute of Medical Sciences and Research Centre

Amrita VishwaVidyapeetham

Kerala, INDIA

Corresponding Author*

Dr Krishnakumar N Menon

Center for Nanosciences and Molecular Medicine

Amrita Vishwa Vidyapeetham

Ponekkara, Kochi-682041

krishnakumarmenon@aims.amrita.edu

Supplementary Information

Figures and Tables

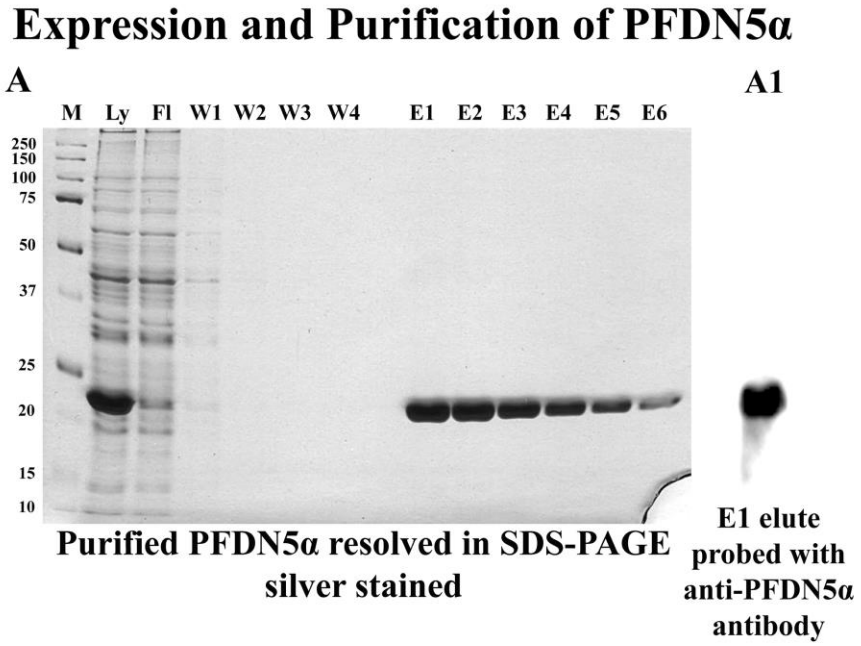

Supplementary Figure S1: Expression and purification of recombinant PFDN5α. Recombinant PFDN5α was expressed in bacteria and purified using Ni-NTA beads as per manufacturer's protocol. The lysate and different fractions during purification procedure (flow through, washes and elutions) were resolved on SDS-PAGE and stained with silver (A). Note the high purity of the proteins obtained. The expressed proteins were finally validated by probing with antibody specific to the proteins following western blotting (A1). M-Protein marker, Ly-Lysate, Fl-Flow through, W1, W2, etc. -Washes and E1, E2, etc.-Elutions.

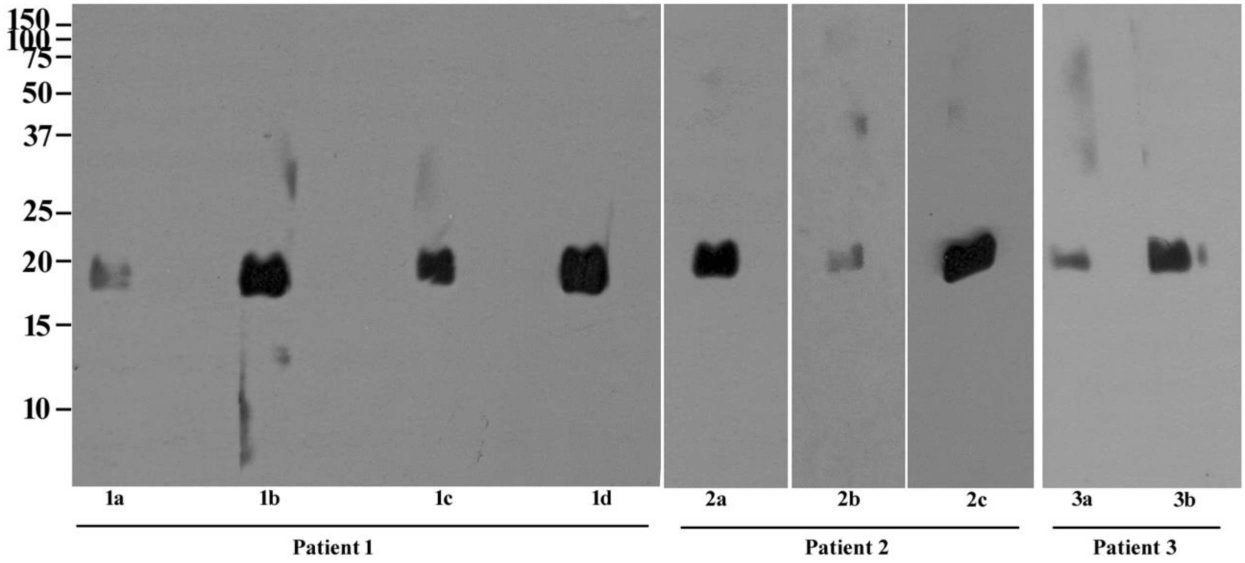

Supplementary Figure S2. Details of the western blot shown in Fig 2A depicting the differential CSF reactivity of CNS+ve (presentation/relapse) and CNS–ve (remission) sequential samples from patients 1, 2 and 3 towards purified recombinant PFDN5α. The pattern of CSF reactivity validates to that observed on the 2D far western blot (Figure 1A). Each lane following western blotting of purified PFDN5-α is cut into single strips. Subsequently, the individual strips were probed with biotinylated individual CSF samples collected at different time points from the same patients (sequential CSF samples of the patient). 1a, 2b and 3a are relapse showing CNS positivity and the rest are CSF samples at remission from the respective three patients.

Supplementary Tables

Supplementary Table S1: The demographic details of the patient samples used for 2D far-western analysis and ELISA quantification of reactivity. Patients were classified as CNS positive (at presentation/diagnosis or as relapse) and CNS negative based on the cytological analysis of CSF samples in the hospital. Samples marked with *indicates that these samples were not available for ELISA quantification due to shortage in quantity required for analysis following 2D far western analysis and 1D western analysis.

**Patient No**	**Age/ Sex**	**Sequential CSF samples collected for analysis**	**CSF samples used for 2D-PAGE far-western and 1D western analysis**	**CSF samples used for ELISA**	**% Blasts in Blood at diagnosis**	**% Blasts in Bone marrow at diagnosis**	**CNS status by CSF Cytology**	**Cytogenetics**	**Treatment Protocol**
**1**	33/M	1a (Diagnosis)	+	+	78	96	CNS+ve Atypical cells present	Normal	BFM 90 modified protocol and EBRT-2400cGy in 12 fractions
		1b (3 weeks after 1a)*	+	-			CNS-ve No atypical cells present		
		1c (4 weeks after 1a)*	+	-			CNS-ve No atypical cells present		
		1d (4 months after 1a)*	+	-			CNS-ve No atypical cells present		
**2**	45/F	2a (3 months after diagnosis)*	+	-	16	60	CNS-ve No atypical cells present	BCR-ABL+ve	BFM 90 protocol and EBRT-1800cGy in 10 fractions
		2b (1year 3 months after 2a)	+	+			CNS+ve Atypical cells present		
		2c (1year 4.5 months after 2a)*	+	-			CNS-ve No atypical cells present		

**3**	8/M	3a (5 years after diagnosis)	+	+	Data not available	50	CNS+ve Atypical cells present	Normal	BFM 86 Relapse protocol
		3b (2 months after 3a)*	+	-			CNS-ve No atypical cells present		
		3c (5.5 months after 3a)*	+	-			CNS-ve No atypical cells present		
**4**	5/M	4a (2.5years after diagnosis)	-	+	Data not available	14	CNS+ve Atypical cells present	Normal	BFM 85, BFM 85 relapse protocol & BFM 84 REZ protocol
		4b (2 weeks after 4a)	-	+			CNS+ve Atypical cells present		
		4c (3 months after 4a)	-	+			CNS+ve Atypical cells present		
		4d (9 months after 4a)	-	+			CNS-ve No atypical cells present		
		4e (11.5 months after 4a)	-	+			CNS+ve Atypical cells present		
		4f (1year after 4a)	-	+			CNS-ve No atypical cells present		
		4g (1year 2 months after 4a)	-	+			CNS-ve No atypical cells present		
		4h (1year 4 month after 4a)	-	+			CNS+ve Atypical cells present		

		4i (1.5years after 4a)	-	+			CNS+ve Atypical cells present		
		4j (1year 7 month after 4a)	-	+			CNS+ve Atypical cells present		
		4k (1year 8 month after 4a)	-	+			CNS+ve Atypical cells present		
		4l (1year 8.5month after 4a)	-	+			CNS+ve Atypical cells present		
		4m (1year 9 months after 4a)	-	+			CNS+ve Atypical cells present		
		4n (1year 9.5 months after 4a)	-	+			CNS-ve No atypical cells present		
		4o (1year 10 months after 4a)	-	+			CNS-ve No atypical cells present		
		4p (1year 10.5 months after 4a)	-	+			CNS+ve Atypical cells present		
		4q (1year 11 months after 4a)	-	+			CNS+ve Atypical cells present		
**5**	2/M	5a (1year 7 months after diagnosis)	-	+	8	97	CNS-ve No atypical cells present	Normal	Pediatric BFM 95 protocol & REZ 85 relapse protocol
		5b (1 month after 5a)	-	+			CNS+ve Atypical cells present		
		5c (8.5 months after 5a)	-	+			CNS+ve Atypical cells present		

**6**	19/M	6a (Diagnosis)	-	+	27	6	CNS+ve Atypical cells present	Normal	Modified BFM 95 Protocol
		6b (2.5 months after 6a)	-	+			CNS+ve Atypical cells present		
		6c (4.5 months after 6a)	-	+			CNS-ve No atypical cells present		
		6d (1year 5.5 months after 6a)	-	+			CNS-ve No atypical cells present		
		6e (1year 7.5 months after 6a)	-	+			CNS-ve No atypical cells present		
**7**	4/M	7a (3years after diagnosis)	-	+	Data not available	92	CNS-ve No atypical cells present	Normal	BFM 95 & BFM 95 relapse protocol
		7b (1year two weeks after 7a)	-	+			CNS+ve Atypical cells present		
		7c (1year 1 month after 7a)	-	+			CNS+ve Atypical cells present		
		7d (1year 1.5 months after 7a)	-	+			CNS-ve No atypical cells present		
		7e (1year 3.5 months after 7a)	-	+			CNS-ve No atypical cells present		
		7f (1year 4.5 months after 7a)	-	+			CNS-ve No atypical cells present		

		7g (1year 6.5 months after 7a)	-	+			CNS-ve No atypical cells present		
		7h (1year 7.5 months after 7a)	-	+			CNS-ve No atypical cells present		
		7i (1year 10 months after 7a)	-	+			CNS-ve No atypical cells present		
		7j (1year 11 months after 7a)	-	+			CNS-ve No atypical cells present		
**8**	4/F	8a (Diagnosis)	-	+	27	96	CNS+ve Atypical cells present	Normal	BFM ALL 2009 Protocol
**9**	8/M	9a (7years after diagnosis)	-	+	9	85	CNS+ve Atypical cells present	Normal	BFM 95 protocol
		9b (6 months after 9a)	-	+			CNS-ve No atypical cells present		
**10**	6/F	10a (4years after diagnosis)	-	+	40	99	CNS+ve Atypical cells present	Normal	BFM 85 relapse protocol
**11**	23/M	11a (6years after diagnosis)	-	+	Data not available	90	CNS+ve Atypical cells present	Normal	BFM 90 relapse protocol
**12**	6/F	12a (1year after diagnosis)	-	+	6	84	CNS-ve No atypical cells present	del(9)(q), aberrant CD 117	Pediatric BFM 95 Protocol
**13**	34/M	13a (1 month after diagnosis)	-	+	7	95	CNS-ve No atypical cells present	Normal	BFM 95 Protocol

		13b (1 month after 13a)	-	+			CNS-ve No atypical cells present		
**14**	4/M	14a (1year 5 months after diagnosis)	-	+	Data not available	96	CNS-ve No atypical cells present	Normal	BFM 95 Protocol
**15**	6/F	15a (2 weeks after diagnosis)	-	+	34	70	CNS-ve No atypical cells present	Normal	Pediatric BFM 95 Protocol
**16**	3/F	16a (2 months after diagnosis)	-	+	Data not available	85	CNS-ve No atypical cells present	Normal	BFM 95 Protocol
**17**	45/M	17a (2 months after diagnosis)	-	+	23	38	CNS-ve No atypical cells present	Aberrant CD33	G MALL protocol
**18**	5/F	18a (1.5 years after diagnosis)	-	+	Data not available	43	CNS-ve No atypical cells present	Normal	Pediatric BFM 95 Protocol
**19**	40/M	19a (5 months after diagnosis)	-	+	Data not available	96	CNS-ve No atypical cells present	Normal	Modified G MALL protocol
**20**	31/F	20a (7 months 3 weeks after diagnosis)	-	+	85	84	CNS-ve No atypical cells present	BCR-ABL+ve, Aberrant CD13	BFM 95 Protocol
**21**	5/F	21a (2 months 1 week after diagnosis)	-	+	Data not available	89	CNS-ve No atypical cells present	MLL rearrangement	BFM ALL 2009 Protocol
**22**	6/M	22a (2 months 1 week after diagnosis)	-	+	Data not available	56	CNS-ve No atypical cells present	BCR-ABL+ve & Aberrant CD13	BFM ALL 2009 Protocol
		22b (2months after 22a)	-	+			CNS-ve No atypical cells		

							present		
**23**	18/F	23a (8 months after diagnosis)	-	+	3	42	CNS-ve No atypical cells present	Normal	BFM ALL 2009 Protocol
**24**	2/M	24a (1year 2.5 months after diagnosis)	-	+	26	98	CNS-ve No atypical cells present	Normal	BFM 95 Protocol
**25**	38/M	25a (4 months 1 week after diagnosis)	-	+	Data not available	75	CNS-ve No atypical cells present	19p13.3 rearrangement	G MALL protocol
**26**	4/F	26a (6 months after diagnosis)	-	+	25	96	CNS-ve No atypical cells present	Aberrant CD13 & CD117	BFM ALL 2009 Protocol
**27**	17/M	27a (2 months 2 weeks after diagnosis)	-	+	13	95	CNS-ve No atypical cells present	MLL rearrangement	BFM ALL 2009 Protocol
**28**	3/M	28a (1year 1week after diagnosis)	-	+	Data not available	21	CNS-ve No atypical cells present	Normal	BFM ALL 2009 Protocol
**29**	17/F	29a (3 months 1 week after diagnosis)	-	+	74	98	CNS-ve No atypical cells present	TCF3 rearrangement	BFM ALL 2009 Protocol
**30**	16/F	30a (2 weeks after diagnosis)	-	+	Data not available	96	CNS-ve No atypical cells present	aberrant T cell markers	BFM ALL 2009 Protocol
**31**	6/F	31a (3 months 1 week after diagnosis)	-	+	Data not available	94	CNS-ve No atypical cells present	aberrant CD13	BFM ALL 2009 Protocol

CSF-Cerebrospinal fluid, CNS-Central nervous system, BFM-Berlin-Frankfurt-Münster, EBRT-external brain radiotherapy

Supplementary Table S2: Protein ID of spots 1-4 by mass spectrometric analysis. Spots which showed differential reactivity to CSF samples from B-ALL patients at CNS positivity and at remission were cut out from the equivalent Coomassie stained gel, trypsin digested and analysed by mass spectrometry to decipher the protein ID. The analysis was carried out by ESI Q-TOF mass spectrometry[a].

Spot number on blot	Protein name	Mascot score	Accession number	Sequence coverage %	No. of Unique Peptides	Molecular weight
**1**	Prefoldin subunit 5α (PFDN5α)	92	IPI00015361.1	17%	3	17.3
**2**	Cytokine inducible protein (CIP29)	502	IPI01015600.1	41.33%	5	29.0
**3**	Delta(3,5)-Delta(2,4)-dienoyl-CoA isomerase, mitochondrial (ECH1)	1402	IPI00011416.2	37.20%	8	35.79
**4**	Peroxiredoxin-6 (PRDX6)	957	IPI00220301.5	48.66%	10	25

[a]Individual ions scores > 25 indicate identity or extensive homology ($p<0.05$)

Supplementary Table S3: Functions and reported role in cancer of mass spectrometrically identified proteins.

Proteins	Functions and reported roles of identified proteins in cancer
**PFDN5α**	• A role in hematopoiesis and leukemia pathogenesis, PFDN5α suppresses transcriptional activity of c-Myc by binding to c-Myc's N-terminal region [1,2]<br>• Recruits ubiquitin ligase complex to c-Myc for its degradation mediated by proteasome [3]<br>• Inhibits Wnt4 expression resulting in the suppression of c-Myc expression [4]<br>• PFDN5α interacts with p73α (a cell cycle arrest and apoptosis inducing protein) and c-Myc to prevent the c-Myc mediated suppression of p73α [5]. Roughly, in 35% of acute lymphoblastic leukemia (ALL) and one third of non-Hodgkin lymphomas (NHLs), p73α is disabled [6]<br>• Since c-Myc is an oncogene and its activity is shown in the leukemia pathogenesis [7], the role of PFDN5α in controlling c-Myc activity is crucial in leukemia<br>• Prefoldin subunits (PFDN1-6) has been found to serve as poor prognostic markers in gastric cancer [8]<br>• PFDN5α promotes cell migration via facilitating filopodia formation [9]<br>• A member of molecular chaperone complex involved in stabilizing newly synthesized polypeptides [10]

Supplementary Table S4: ELISA Quantified data of reactivity to PFDN5α to CNS positive and CNS negative CSF samples from B-ALL patients. CNS +ve and –ve samples were selected based on CSF cytology analysis done in the clinic.

CNS Positive Samples	OD	CNS Negative Samples	OD
1	0.132	1	0.673
2	0.099	2	0.462
3	0.321	3	0.275
4	0.044	4	0.44
5	0.179	5	0.803
6	0.041	6	0.413
7	0.213	7	0.39
8	0.314	8	0.943
9	0.018	9	0.407
10	0.2	10	0.487
11	0.357	11	0.861
12	0.205	12	0.759
13	0.019	13	0.619
14	0.246	14	0.703
15	0.008	15	0.722
16	0.64	16	0.629
17	0.004	17	0.669
18	0.453	18	0.249
19	0.517	19	0.323
20	0.028	20	0.374
21	0.024	21	0.323
22	0.454	22	0.355
23	0.055	23	0.469
24	0.631	24	0.335
25	0.417	25	0.265
		26	0.568
		27	0.458
		28	0.471
		29	0.431
		30	0.35
		31	0.306
		32	0.329
		33	0.351
		34	0.475
		35	0.363
		36	0.514
		37	0.647
		38	0.532
		39	0.688
		40	0.645
**Avg**	**0.225**	**Avg**	**0.502**

Supplementary Table S4A: Triplicates of average CSF reactivity value to PFDN5α from CNS positive samples and its average shown in Supplementary Table S4.

CNS positive samples

Sl No	1	2	3	4	5
Value 1	0.13	0.1	0.354	0.058	0.156
Value 2	0.129	0.121	0.27	0.041	0.149
Value 3	0.136	0.076	0.34	0.033	0.232
**Avg**	**0.132**	**0.099**	**0.321**	**0.044**	**0.179**

Sl No	6	7	8	9	10
Value 1	0.035	0.217	0.287	0.040	0.174
Value 2	0.044	0.196	0.354	0.009	0.205
Value 3	0.044	0.227	0.3	0.006	0.22
**Avg**	**0.041**	**0.213**	**0.314**	**0.018**	**0.200**

Sl No	11	12	13	14	15
Value 1	0.367	0.259	0.024	0.256	0.008
Value 2	0.342	0.178	0.02	0.243	0.007
Value 3	0.361	0.177	0.012	0.239	0.009
**Avg**	**0.357**	**0.205**	**0.019**	**0.246**	**0.008**

Sl No	16	17	18	19	20
Value 1	0.72	0.001	0.482	0.517	0.027
Value 2	0.613	0.002	0.405	0.525	0.034
Value 3	0.587	0.009	0.473	0.51	0.022
**Avg**	**0.64**	**0.004**	**0.453**	**0.517**	**0.028**

Sl No	21	22	23	24	25
Value 1	0.021	0.433	0.065	0.597	0.461
Value 2	0.027	0.427	0.064	0.662	0.345
Value 3	0.024	0.501	0.036	0.635	0.446
**Avg**	**0.024**	**0.454**	**0.055**	**0.631**	**0.417**

Sl No	OD
1	0.132
2	0.099
3	0.321
4	0.044
5	0.179
6	0.041
7	0.213
8	0.314
9	0.018
10	0.2
11	0.357
12	0.205
13	0.019
14	0.246
15	0.008
16	0.64
17	0.004
18	0.453
19	0.517
20	0.028
21	0.024
22	0.454
23	0.055
24	0.631
25	0.417
**Avg**	**0.225**

Supplementary Table S4B: Triplicates of CSF reactivity value to PFDN5α from CNS negative patient samples and its average shown in Supplementary Table S4.

CNS Negative samples

**Sl No**	**1**	**2**	**3**	**4**	**5**	**6**	**7**	**8**	**9**	**10**
Value 1	0.654	0.431	0.263	0.425	-	0.43	0.355	0.935	0.431	0.48
Value 2	0.645	0.506	0.255	0.443	0.806	0.42	0.345	0.969	0.421	0.474
Value 3	0.721	0.45	0.308	0.451	0.799	0.388	0.47	0.924	0.369	0.506
**Avg**	**0.673**	**0.462**	**0.275**	**0.440**	**0.803**	**0.413**	**0.390**	**0.943**	**0.407**	**0.487**

**Sl No**	**11**	**12**	**13**	**14**	**15**	**16**	**17**	**18**	**19**	**20**
Value 1	0.811	0.742	0.658	0.819	0.706	0.559	0.692	0.264	0.312	0.378
Value 2	0.856	0.767	0.646	0.736	0.659	0.679	0.671	0.232	0.304	0.376
Value 3	0.916	0.767	0.552	0.553	0.802	0.65	0.643	0.252	0.353	0.367
**Avg**	**0.861**	**0.759**	**0.619**	**0.703**	**0.722**	**0.629**	**0.669**	**0.249**	**0.323**	**0.374**

**Sl No**	**21**	**22**	**23**	**24**	**25**	**26**	**27**	**28**	**29**	**30**
Value 1	0.303	0.284	0.448	0.319	0.249	0.616	0.513	0.524	0.468	0.392
Value 2	0.327	0.391	0.501	0.353	0.261	0.593	0.474	0.469	0.445	0.346
Value 3	0.339	0.39	0.457	0.332	0.285	0.496	0.388	0.42	0.38	0.311
**Avg**	**0.323**	**0.355**	**0.469**	**0.335**	**0.265**	**0.568**	**0.458**	**0.471**	**0.431**	**0.350**

**Sl No**	**31**	**32**	**33**	**34**	**35**	**36**	**37**	**38**	**39**	**40**
Value 1	0.335	0.382	0.381	0.503	0.411	0.554	0.624	0.599	0.769	0.631
Value 2	0.316	0.343	0.368	0.389	0.326	0.52	0.653	0.501	0.723	0.734
Value 3	0.268	0.261	0.305	0.532	0.352	0.469	0.665	0.497	0.572	0.569
**Avg**	**0.306**	**0.329**	**0.351**	**0.475**	**0.363**	**0.514**	**0.647**	**0.532**	**0.688**	**0.645**

**Sl No**	**OD**
1	0.673
2	0.462
3	0.275
4	0.44
5	0.803
6	0.413
7	0.39
8	0.943
9	0.407
10	0.487
11	0.861
12	0.759
13	0.619
14	0.703
15	0.722
16	0.629
17	0.669
18	0.249
19	0.323
20	0.374
21	0.323
22	0.355
23	0.469
24	0.335
25	0.265
26	0.568
27	0.458
28	0.471
29	0.431
30	0.35
31	0.306
32	0.329

33	0.351
34	0.475
35	0.363
36	0.514
37	0.647
38	0.532
39	0.688
40	0.645
**Avg**	**0.502**

Supplementary Table S5: Evaluation of reactivity to PFDN5α of CNS +ve and CNS -ve CSF samples of B-ALL patients by ROC analysis.

	**AUC (95%CI)**	***p*-value**	**Sensitivity (%)**	**Specificity (%)**	**PPV (%)**	**NPV (%)**	**Cut off Value**
**Reactivity to PFDN5α CNS +ve vs CNS -ve**	0.84 (0.74 to 0.95)	<0.0001	88	55	55	88	0.456

AUC-Area under curve, CI-Confidence Interval, PPV-positive predictive value, NPV-Negative predictive value

Supplementary Table S6: Flow cytometric analysis of B-ALL patient samples (negative as per CSF cytology) showing the % of CD34+ and CD19+ cells and the ELISA based quantification of PFDN5-α CSF reactivity of the respective CSF samples analyzed for flow cytometry

CSF Samples from B-ALL patients	Total number of Cells in B-ALL CSF by Flow analysis	Total CD19+/CD 34+ cells in B-ALL CSF	% of blast cells	Average ± SE	CNS Leukemia category	CSF reactivity by ELISA (OD)	Average ± SE
1	1006	1	0.0	0.306 ± 0.160 (Less than 1% cells)	Negative	0.516	0.656 ± 0.075
2	9829	37	0.376		Negative	0.776	
3	10307	56	0.543		Negative	0.676	
4	13411	194	1.446	1.967 ± 0.394 (Less than 3% cells)	Positive	0.604	0.509 ± 0.066
5	641	11	1.716		Positive	0.541	
6	1387	38	2.739		Positive	0.381	
7	1162	37	3.184	11.379 ± 5.444 (Greater than 3% cells)	Positive	0.231	0.448 ± 0.092
8	1547	53	3.425		Positive	0.403	
9	2780	102	3.669		Positive	0.551	
10	580	35	6.034		Positive	0.481	
11	508	37	7.283		Positive	0.315	
12	611	52	8.510		Positive	0.664	
13	1113	118	10.601		Positive	0.559	
14	283	30	10.600		Positive	0.196	
15	574	85	14.808		Positive	0.674	
16	272	65	23.897		Positive	0.383	
17	187	62	33.155		Positive	0.468	

References

1. Mori, K.; Maeda, Y.; Kitaura, H.; Taira, T.; Iguchi-Ariga, S.M.M.; Ariga, H. MM-1, a novel c-Myc-associating protein that represses transcriptional activity of c-Myc. *J. Biol. Chem.* **1998**, *273*, 29794–29800.

2. Satou, A.; Taira, T.; Iguchi-Ariga, S.M.M.; Ariga, H. A novel transrepression pathway of c-Myc: Recruitment of a transcriptional corepressor complex to c-Myc by MM-1, a c-Myc-binding protein. *J. Biol. Chem.* **2001**, *276*, 46562–46567.

3. Kimura, Y.; Nagao, A.; Fujioka, Y.; Satou, A.; Taira, T.; Iguchi-Ariga, S.M.M.; Ariga, H. MM-1 facilitates degradation of c-Myc by recruiting proteasome and a novel ubiquitin E3 ligase. *Int. J. Oncol.* **2007**, *31*, 829–836.

4. Yoshida, T.; Kitaura, H.; Hagio, Y.; Sato, T.; Iguchi-Ariga, S.M.M.; Ariga, H. Negative regulation of the Wnt signal by MM-1 through inhibiting expression of the wnt4 gene. *Exp. Cell Res.* **2008**, *314*, 1217–1228.

5. Watanabe, K.I.; Ozaki, T.; Nakagawa, T.; Miyazaki, K.; Takahashi, M.; Hosoda, M.; Hayashi, S.; Todo, S.; Nakagawara, A. Physical interaction of p73 with c-Myc and MM1, a c-Myc-binding protein, and modulation of the p73 function. *J. Biol. Chem.* **2002**, *277*, 15113–15123.

6. Corn, P.G.; Kuerbitz, S.J.; Van Noesel, M.M.; Esteller, M.; Compitello, N.; Baylin, S.B.; Herman, J.G. Transcriptional silencing of the p73 gene in acute lymphoblastic leukemia and Burkitt's lymphoma is associated with 5' CpG island methylation. *Cancer Res.* **1999**, *59*, 3352–3356.

7. Fujioka, Y.; Taira, T.; Maeda, Y.; Tanaka, S.; Nishihara, H.; Iguchi-Ariga, S.M.M.; Nagashima, K.; Ariga, H. NM-1, a c-Myc-binding Protein, Is a Candidate for a Tumor Suppressor in Leukemia/Lymphoma and Tongue Cancer. *J. Biol. Chem.* **2001**, *276*, 45137–45144.

8. Yesseyeva, G.; Aikemu, B.; Hong, H.; Yu, C.; Dong, F.; Sun, J.; Zang, L.; Zheng, M.; Ma, J. Prefoldin subunits (PFDN1-6) serve as poor prognostic markers in gastric cancer. *Biosci. Rep.* **2020**, *40*, 1–11.

9. Fan, S.; Chen, Y.; Jiang, Y.; Hu, K.; Li, C. Prefoldin subunit MM1 promotes cell migration via facilitating filopodia formation. *Biochem. Biophys. Res. Commun.* **2020**,

533, 613–619.

10. Vainberg, I.E.; Lewis, S.A.; Rommelaere, H.; Ampe, C.; Vandekerckhove, J.; Klein, H.L.; Cowan, N.J. Prefoldin, a chaperone that delivers unfolded proteins to cytosolic chaperonin. *Cell* **1998**, *93*, 863–873.
